# Temporal Validation of an FDG-PET-Radiomic Model for Distant-Relapse-Free-Survival After Radio-Chemotherapy for Pancreatic Adenocarcinoma

**DOI:** 10.3390/cancers17061036

**Published:** 2025-03-20

**Authors:** Monica Maria Vincenzi, Martina Mori, Paolo Passoni, Roberta Tummineri, Najla Slim, Martina Midulla, Gabriele Palazzo, Alfonso Belardo, Emiliano Spezi, Maria Picchio, Michele Reni, Arturo Chiti, Antonella del Vecchio, Claudio Fiorino, Nadia Gisella Di Muzio

**Affiliations:** 1 Medical Physics, IRCCS San Raffaele Scientific Institute, 20132 Milan, Italy; vincenzi.monica@hsr.it (M.M.V.);; 2 Radiotherapy, IRCCS San Raffaele Scientific Institute, 20132 Milan, Italy; 3 School of Engineering, Cardiff University, Cardiff CF24 4HQ, UK; 4 Nuclear Medicine, IRCCS San Raffaele Scientific Institute, 20132 Milan, Italy; 5 Department of Medical Oncology, Faculty of Medicine and Surgery, Vita-Salute University, 20132 Milan, Italy; 6 Oncology, IRCCS San Raffaele Scientific Institute, 20132 Milan, Italy; 7 Department of Imaging Diagnostics, Neuroradiology, and Radiotherapy, Faculty of Medicine and Surgery, Vita-Salute University, 20132 Milan, Italy

**Keywords:** pancreatic cancer, radiotherapy, radiomic, predictive models, distant relapses, temporal validation

## Abstract

**Background/Objectives**: Pancreatic cancer is a very aggressive disease with a poor prognosis, even when diagnosed at an early stage. This study aimed to validate and refine a radiomic-based [^18^F]FDG-PET model to predict distant relapse-free survival (DRFS) in patients with unresectable locally advanced pancreatic cancer (LAPC). **Methods**: A Cox regression model incorporating two radiomic features (RFs) and cancer stage (III vs. IV) was temporally validated using a larger cohort (215 patients treated between 2005–2022). Patients received concurrent chemoradiotherapy with capecitabine and hypo-fractionated Intensity Modulated Radiotherapy (IMRT). Data were split into training (145 patients, 2005–2017) and validation (70 patients, 2017–2022) groups. Seventy-eight RFs were extracted, harmonized, and analyzed using machine learning to develop refined models. **Results**: The model incorporating Statistical-Percentile10, Morphological-ComShift, and stage demonstrated moderate predictive accuracy (training: C-index = 0.632; validation: C-index = 0.590). When simplified to include only Statistical-Percentile10, performance improved slightly in the validation group (C-index = 0.601). Adding GLSZM3D-grayLevelVariance to Statistical-Percentile10, while excluding Morphological-ComShift, further enhanced accuracy (training: C-index = 0.654; validation: C-index = 0.623). Despite these refinements, all versions showed similar moderate ability to stratify patients into risk classes. **Conclusions**: [^18^F]FDG-PET radiomic features are robust predictors of DRFS after chemoradiotherapy in LAPC. Despite moderate performance, these models hold promise for patient risk stratification. Further validation with external cohorts is ongoing.

## Simple Summary

Pancreatic cancer is a highly aggressive disease with a poor prognosis, even when detected in its early stages. This study temporally validated and improved a model using radiomic features derived from [^18^F]FDG-PET imaging to predict distant relapse-free survival in patients with locally advanced pancreatic cancer. Data from 215 patients treated with chemoradiotherapy were analyzed. The original model, which included two radiomic features and a cancer stage, showed moderate accuracy in predicting patient outcomes. Simplifying the model to a single radiomic feature improved performance slightly, while adding another complementary feature further enhanced accuracy. Although all versions of the model showed moderate ability to differentiate risk levels, these radiomic features demonstrate potential for patient stratification. Further validation is ongoing with independent cohorts from external centers, ensuring robustness beyond the analyzed patient group.

## 1. Introduction

Pancreatic adenocarcinoma is one of the most severe cancers in terms of prognosis and ranks as the sixth leading cause of cancer mortality, with increasing incidence in countries with a high human development index [1,2]. Despite some advancements, the 5-year overall survival (OS) rates remain below 10% [3,4]. Although surgery is associated with better survival rates, it is often not feasible due to the advanced stage of the disease or other exclusion criteria [5,6]. Most patients are diagnosed at a locally advanced (LAPC) or metastatic stage, resulting in lower 5-year survival rates [3,4].

Chemoradiotherapy (CRT) is a common treatment, but it has often been associated with risks of severe toxicity and limited survival: typically, median survival of approximately 5–15 months and a 2-year survival rate below 30% were reported, often accompanied by distant relapses [7,8,9,10]. On the other hand, the advancements of Radiotherapy (RT) planning and delivery techniques suggested the possibility to deliver higher doses without increasing toxicity, with the hope to improve loco-regional control [11,12,13,14]. In particular, better image-guided radiotherapy (IGRT) approaches, including the consideration of tumor motion, showed the possibility to apply moderately hypo-fractionated techniques [15,16,17,18] as well as stereotactic body RT [19,20,21,22,23,24,25,26]. Although not yet assessed, results show that there is room for substantial dose escalation, hopefully without increasing toxicity [15,16,17,27,28]. Furthermore, the recent advent of MR-guided RT enhanced this hope [29,30,31,32]. However, the relevant fraction of patients experiencing early distant relapses makes the issue controversial, due to the likely limited or null cost-benefit ratio in applying more aggressive local treatments for these patients. It is out of doubt that local intensification for all patients is not a rational choice, given the large fraction of patients with poor outcomes due to metastatic spread, despite the potential improvement of loco-regional control. However, the lack of reliable models able to identify patients at higher risk of early distant metastatic spread, leaves the issue unsolved. Ideally, the availability of reliable models to predict distant relapse-free survival (DRFS) could significantly enhance treatment personalization for LAPC, permitting the selection of patients who effectively could benefit from more aggressive local RT [33].

Currently, key factors for predicting survival in pancreatic cancer patients include tumor size, grade of differentiation, and lymph node status [34]. Among molecular biomarkers, CA 19-9 (carbohydrate antigen 19.9) is the only FDA-approved marker for LAPC and is useful for treatment monitoring and early detection of recurrences [35,36,37]. However, it is not specific to pancreatic cancer and may be elevated in other conditions, with some patients unable to produce it [34].

The search for reliable imaging biomarkers is a relevant field of research in LAPC even due to their potential widespread availability, low cost, and non-invasiveness. Several studies investigated the potential of CT and PET biomarkers showing promising results [38]. In particular, ^18^F-fluorodeoxyglucose PET (^18^F-FDG-PET) is growingly available for these patients and may provide additional quantitative information to predict tumor behavior, considering SUV_max_ and SUV_mean_ values, metabolic tumor volume (MTV), and total lesion glycolysis (TLG) [39,40].

More advanced analyses in the field of radiomics aim to convert medical images into quantitative descriptors of tumor tissues, providing details on intensity, shape, size, and texture of tumors [41]. PET images are particularly promising since intensity heterogeneity appears related to tumor biology [42,43], though not many studies have explored pancreatic cancer in this context [44,45,46,47,48,49]. However, a critical issue of radiomics concerns its difficulty in being usable outside the training cohort, mainly due to the lack of reliability and repeatability of the radiomic features (RFs) extracted from the PET-based gross target tumor (GTV) for pancreatic cancer [50,51,52,53,54,55,56].

A few years ago, a robust PET-radiomic model to predict DRFS for LPAC patients treated with CRT was developed by our group; rigorous consideration of all potential uncertainties was applied together with a strict methodology for RF selection in building the model. This resulted in a 2-RFs model (here named the Mori index) with high explainability and good performances in predicting the risk of DRFS [57].

To corroborate the strength of such model and/or to refine it, a new study (RADIOMIPa) was started to temporally and externally validate it, including data from external centers. The first part concerned temporal validation, and the current study reports these results. The uniqueness of this study lies precisely in the temporal validation of a radiomic index, an aspect, to our knowledge, never addressed in the existing literature.

The current investigation aims to: (i) validate the Mori index in a more recent and enlarged cohort of patients of our Institute; (ii) refine the Mori model by checking the robustness of each single RF and (iii) consider the addition of new, best-performing, RFs to obtain a fine-tuned index, temporally validated following the same approach.

## 2. Materials and Methods

### 2.1. Patient Population

The following retrospective study enrolled 215 patients with histologically confirmed LAPC, who underwent a PET/CT scan with ^18^F-FDG (resulting positive) between 2005 and 2022 and then received CRT at the San Raffaele Hospital in Milan. The institutional ethics committee approved the retrospective revision of patient outcomes (registration number: 160/INT/2021, dated 13 October 2021), and all patients provide consent for the treatment of their data. All patients were deemed unresectable according to NCCN Guidelines (version 2.2018) and excluded from surgery. Details on the administration of CRT were previously described [15,27,57]. Patients were classified according to the sixth edition of the AJCC pancreatic cancer staging system, in use at the time of patient enrollment of the training data set [58,59]: this system was used also for the validation set, for consistency reasons. In short, patients with stage III and IV cancers who exhibited a complete clinical response after 4 months of induction chemotherapy were treated with CRT, according to institutional policies [60], targeting tumors and PET-positive lymph nodes. As discussed in the original Mori et al. paper [57], the selected grade IV patients were retained in current analyses as they were not submitted to CRT with palliative purpose, receiving the same treatment received by the grade III patients. Given the aims of the current validation study, patients were included following the same criteria of the training data set: then, stage IV patients in complete response after neo-adjuvant chemotherapy were retained. On the other hand, no impact was previously reported on the original Mori model [57] if excluding these patients from the analysis. The induction chemotherapy consisted of four to six cycles of drugs such as cisplatin, epirubicin, 5-fluorouracil or capecitabine, and gemcitabine.

Radiotherapy was administered using IMRT in a moderate hypofractionation approach (44.25 Gy in 15 fractions), with a boost of up to 48–58 Gy for selected patients (n = 60) whose tumors infiltrate the peri-pancreatic vessels [15]. The concomitant chemotherapy included capecitabine at 1250 mg/m^2^ per day.

Patients were monitored every 2 months via contrast-enhanced thoracic and abdominal CT and serum CA 19.9, with an FDG-PET/CT performed approximately 4 months after CRT. Disease progression and death were prospectively recorded. For stage IV patients, the appearance of metastases at a different site was defined as distant relapse.

To perform the temporal validation, the patients were divided into two temporally consecutive groups: a training group (145 patients from June 2005 to September 2017) on which predictive variables were found, and a validation group (70 patients treated from September 2017 to November 2022) on which the found models were tested. The main characteristics of the patients are reported in Table 1.

### 2.2. Image Acquisition and Tumor Segmentation

PET images were all acquired at San Raffaele Hospital using three different scanners (Discovery-ST, Discovery-STE, and Discovery-690, General Electric Medical Systems, Milwaukee, WI, USA). Following an internal protocol, the images were acquired in static emission on average 60 min after tracer injection; the dose of ^18^F-FDG was 370 MBq. Attenuation correction and image co-registration were performed using CT image data.

All images were resampled with cubic voxels of 3 × 3 × 3 mm^3^ using an automatic interpolation available on the commercial software used in the analysis (MIM Software Inc., Cleveland, OH, USA, v. 7.3.2). This resampling followed the recommendations of the International Biomarker Standardization Initiative (IBSI) [61,62,63,64].

Tumors were segmented using a previously validated semi-automatic gradient-based method (PET-Edge, MIM Software Inc.) [50] available in the MIM software.

### 2.3. Extraction of Radiomic Features

The extraction of RFs in accordance with IBSI guidelines [64,65] was performed using the MATLAB (MathWorks, Natick, MA, USA, v. 9.11) version of the Spaarc Pipeline for Automated Analysis and Radiomics Computing (SPAARC 2023, https://spaarc-radiomics.io/, accessed on 11 March 2025) [59], which was automated in-house using Python (v.3.9) scripts.

Within SPAARC, a discretization technique using 64 fixed bins was established according to Tixier et al. [65] and previously validated [64].

A total of 182 first- and higher-order RFs were extracted, belonging to the following families: Morphology, Statistics, Intensity Histogram, 3D Gray-Level Co-occurrence Matrix average (GLCM3D_avg), 3D Gray-Level Co-occurrence Matrix combined (GLCM3D_comb), 3D Gray-Level Run-Length average (GLRL3D_avg), 3D Gray-Level Run-Length combined (GLRL3D_comb), 3D Gray-Level Size Zone Matrix, 3D Neighboring Gray Tone Difference Matrix (NGTDM3D), 3D Gray-Level Distance Zone Matrix (GLDZM3D).

Among all these features, only the 78 RFs that were identified as robust and showed no inter-operator variability in the previous study [57] were selected and included in the models. In the Supplementary Materials (Section S1), detailed lists of all the features considered are provided.

### 2.4. Model Creation

First, the Mori model was retested, looking to its reliability against time. Specifically, the model included two features: the Morphological-COMshift and Statistical-Percentile10. Then, new models were developed to refine the previously found Mori model, following similar methods. Modeling was performed using an in-house-developed machine learning code (medicalAI in mAItre (Medical Artificial Intelligence Toolkit for Research) https://github.com/pymaitre, accessed on 11 March 2025).

To identify the best combination of RFs predictive of DRFS outcomes, the bootstrap technique with 1000 populations was applied to the 145 training patients, as detailed in the Supplementary Materials. Specifically, a correlation filter based on the calculation of the Spearman correlation coefficient was applied to the 78 RFs that were found to be robust with respect to inter-observer variability and intra-scanner differences. By considering only RFs with a Spearman r > 0.80 and a *p*-value less than 0.05, 18 RFs were selected for the bootstrap procedure, as shown in Table S2 of the Supplementary Materials.

Finally, the resulting multi-variate Cox proportional hazards models were run on the training population using MedCalc^®^. (MedCalc Software Ltd., Ostend, Belgium; version 22.006), considering only the RFs found to be predictive. This provided both the prognostic indices (PI) and the coefficients β to assign to the features. From this distribution of PIs, the ROC curve was calculated to obtain the best separation criterion (Youden), which was then applied to derive the corresponding Kaplan–Meier curves.

### 2.5. Model Validation

For the validation population, the previously obtained coefficients *β* were used to apply the equation

(1) $$PI=\sum_{i}\beta_{i}\cdot RF_{i}$$

to obtain the *PI*s. The ROC curve was then calculated to obtain the Youden index, and finally, the Kaplan–Meier separation was derived.

To determine which model was the best between the Mori model and the new ones, both the metrics related to the parameters entering the individual models and the *p*-values of separation were compared. Additionally, the Spearman matrix resulting from medicalAI was analyzed to assess the correlation between the RFs resulting from the various models.

### 2.6. Data Set Harmonization

Since the PET images were acquired using three different scanners, the inter-scanner variability was studied. Specifically, the distributions of the RFs extracted from SPAARC for the three scanners were analyzed and compared using the Mann–Whitney test. Subsequently, the RFs were harmonized using the ComBat method [66,67,68,69]. Afterward, the entire procedure (described in the previous two paragraphs) was repeated to both re-obtain the models and validate them. In particular, the validation was performed on both the new models and the previously obtained models but with the harmonized RFs. Of note, in the original Mori study, the differences between scanners of all RFs were not found significantly different while in the larger, currently considered, cohort, they showed statistically significant differences for 11 out of the 18 RFs considered robust and correlated (see Table S4 for the specific features and scanners involved).

## 3. Results

The training population showed a median follow-up (FU) of 13.04 months (m) (range: 0.35–166.8 m). Within this group, 90 patients (62%) exhibited distant recurrence-free survival (DRFS) with a median time to occurrence of 6.88 m, while 74 patients (51%) showed locoregional recurrence-free survival (LRFS) with a median time of 14.9 m.

For the validation cohort, the median FU was 15.33 m (range: 0.71–59.29 m). In this population, 41 patients (59%) achieved DRFS with a median time to occurrence of 7.52 m, and 35 patients (50%) showed LRFS with a median time of 14.92 m.

### 3.1. Mori Model Validation

A general overview of the models considered is provided in the Supplementary Materials (Section S3). Concerning the Mori model, based on the two RFs included, the prognostic indices (PI) were calculated for the training population (median PI = 0.64 [−1.18; 2.32]; *p*-value = 0.0009; HR = 1.85), and the validation population (median PI = 0.68 [−0.40; 1.99]; *p*-value = 0.12; HR = 1.72). The temporal validation confirmed in part the original performances (in terms of HR) without reaching statistical significance. The improvement of this model is confirmed with performance comparable to that obtained by Mori et al. [57], even with the addition of 40 patients treated consecutively over time. This confirms the robustness of the RFs over time.

However, as shown in Table 2, the RF driving this model was found to be Statistical-Percentile 10. When considering only this RF (Model 2), results were much improved, both for the training cohort (median PI = 0.94 [0.49; 2.51]; *p*-value = 0.0011; HR = 2.53), and the validation cohort (median PI = 0.96 [0.74; 23.03]; *p*-value = 0.0522; HR = 1.7).

### 3.2. Fine-Tuned Models Validation

The best-performing model (Model 3) included two variables: Statistical minGreyLevel and Intensity Histogram coefficient of Variation. These two RFs were significantly correlated with the original Mori model, showing a Spearman r-coefficient equal to −0.25 and 0.3 for COMshift and 0.91 and 0.21 for Percentile10, respectively. This fine-tuned model proved to perform better than the Mori model, as evidenced by Table 2 (Training: median PI = −0.93 [−2.91; 0.81]; *p*-value = 0.0008; HR = 2.83. Validation: median PI = −0.96 [−2.62; 0.2]; *p*-value = 0.021; HR = 1.87).

### 3.3. Model After Harmonization

Following harmonization using the ComBat method, the previously presented models were reanalyzed. As shown in Table 2, the models showed good performances, with some improvement after harmonization.

Subsequently, the MedicalAI code was run again on the RFs harmonized with the bootstrap method. The Statistical-Percentile10 feature remains predictive in the univariate model, with a *p*-value of 5.72 × 10^−4^. A new two-variable model was identified (Model 4), where the selected RFs were Statistical-Percentile10 and GLSZM3D-grayLevelVariance (anticorrelated with COMshift at −0.24), as detailed in Table 2.

**Figure 1:**
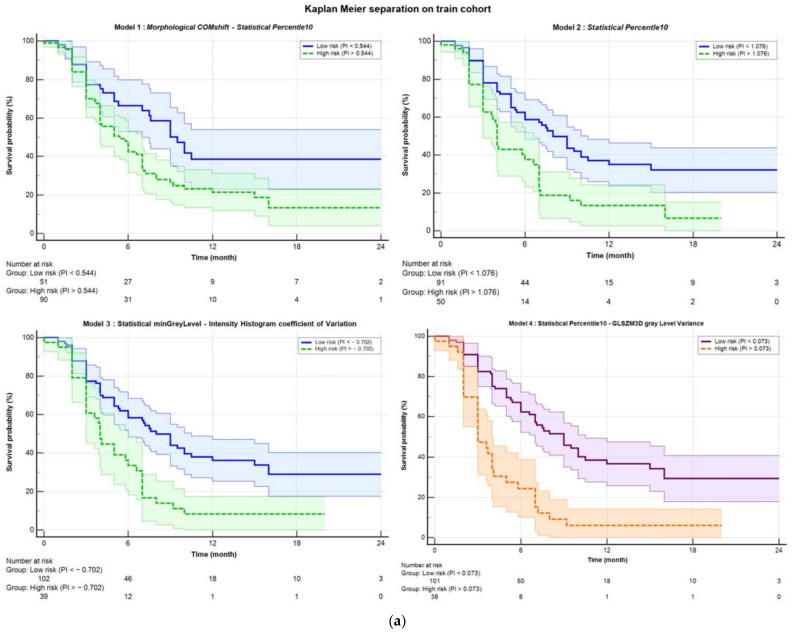

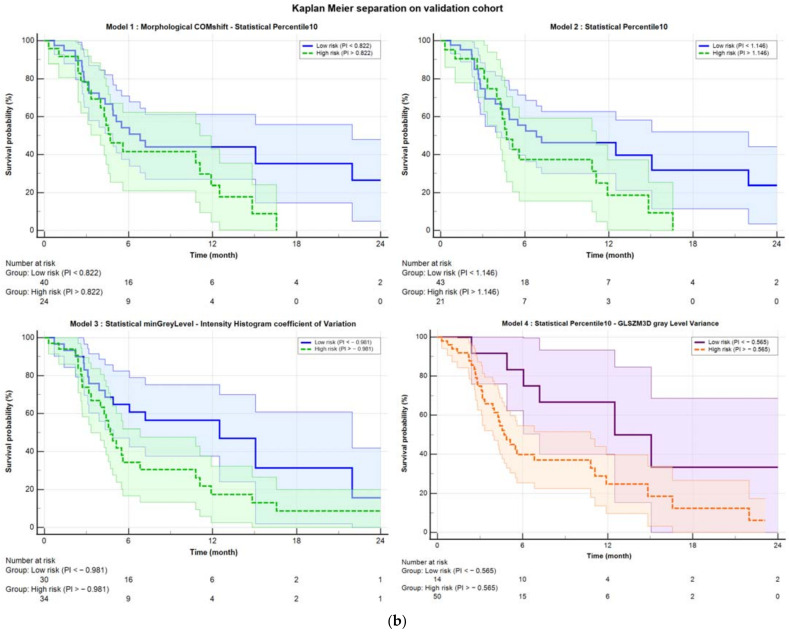
Kaplan–Meier separation curves for (**a**) training and (**b**) validation cohort with corresponding confidence bands. The solid blue line represents the curve for low probability, while the dashed green line represents the high-risk curve. The separation was performed based on the Youden index obtained from ROC analysis. Starting from the top left, the separations are shown for Model 1 (COMshift and Percentile10), Model 2 (Percentile10), and Model 3 (minGrayLevel and coefficient of Variation). In the bottom right, Model 4 is displayed, which was obtained after harmonization using the ComBat method. The purple solid line represents the low-risk curve, and the dashed orange line represents the high-risk curve, both with their respective confidence bands.

Table 2 shows the performances of all four models: the original Mori model (Model 1), the one-variable model (Percentile10, Model 2), and the best fine-tuned models before (Model 3) and after (Model 4) harmonization. Figure 1 illustrates the ability of the corresponding PI to stratify patients in high and low risk of DRFS in both the training (Figure 1a) and the validation cohorts (Figure 1b). As evidenced by the *p*-values from the Logrank test (“KM p” column in Table 2), which measures separation capability, Model 4 showed a slightly better performance, with a *p*-value of 0.0001 in the training cohort and 0.028 in the validation cohort.

### 3.4. Addition of Grading

As evidenced by the paper by Mori et al. [57], the only clinical variable impacting the models was previously found “stage IV” (vs III). Therefore, the analysis was repeated by adding this variable as a predictive factor. Specifically, binary values were assigned, with 0 representing stage III and 1 representing stage IV (16 patients in the training set and 10 patients in the validation set). The results of this analysis are presented in Table 3.

Figure S2 in the Supplementary Materials illustrates the ability of the prognostic index to stratify patients in both the training cohort (Figure S2a) and the validation cohort (Figure S2b) after adding the clinical variable of stage (IV vs. III). In general, the performances of the models slightly improved in terms of C-index and HR, confirming a moderate independent impact of grading on the models’ performances.

## 4. Discussion

This study aimed to temporally validate a radiomic model based on robust features to predict distant recurrence-free survival (DRFS). The analysis was conducted on data from 215 LAPC patients, including 26 complete responding patients in stage IV, treated with induction chemotherapy followed by chemoradiotherapy, according to an institutional protocol involving moderate hypofractionation.

To our knowledge, this is the first time that a radiomic predictive model based on consistent and robust features extracted from PET images for LAPC has undergone temporal validation. While several studies address radiomic models, they typically involve CT analysis [70,71], sometimes combining it with genomics, biomarkers, and clinical data [72,73,74], or on other tumor types, such as pancreatic ductal adenocarcinoma [75,76,77,78,79,80,81,82,83]. Other studies have highlighted the predictive value of PET radiomics in the diagnosis and prognosis of PDAC [56,84]. Other studies that include PET radiomics and biomarkers achieve, to our knowledge, performance comparable to those of this work [45,46,47]. On the other hand, none of them were submitted to any temporal validation.

The study follows previous investigations that were dedicated to the implementation and refinement of a robust and IBSI consistent pipeline for hand-crafted radiomic analyses [50,57,64,71,81,85] for CT and PET features. These studies permitted to first identify the more stable PET features for LAPC when considering their repeatability and inter-observer variability [50,64], skipping those more unstable from the analysis. Additionally, the use of a validated semi-automatic segmentation method using commercial software and of an IBSI-compliant software for RF extraction strengthens the validity of the methodology. A first model was trained, a few years ago, and internally validated by Mori et al. [57]: the model was based on only two, first-order, independent radiomic features (RFs); this current study dealt with the question concerning the possibility of confirming the stability of this model against the time.

As is known, changes in clinical practice and patient characteristics, changing use of available scanners and image-acquisition protocols, and other unknown biases may interfere with the possibility of replicating with time the performances of a radiomic predictive model; although this is a relevant issue, it has been seldom investigated and never, to our knowledge, in the case of PET radiomic for LAPC outcome prediction.

For this reason, the previously investigated cohort was reconsidered and enlarged with the patients treated after the conclusion of the first Mori study. The resulting cohort was much larger and was then divided into training (145 patients) and validation (70 patients) assessed in a temporal manner, using only the date of PET imaging as a reference.

The aim of the study was not only to test the Mori model performances but also to possibly further refine the analysis by re-running the feature-selection procedures and by harmonizing the features using the ComBat method [66,67,68,69]. The results were positive, showing a moderate replicability of the Mori model (once corrected for stage) with time, more pronounced if harmonization is applied. In addition, the evidence that one of the two originally selected features (Percentile10) was much more robust than the other one (COMShift), showed that a model with only Percentile10 (named Model 2) could work similarly well. As a matter of fact, Model 2 showed slightly better performance than the original model, confirming that Model 1 is driven mainly by Percentile10. Interestingly, the newly refined models showed slightly improved performances and confirmed the validity of Percentile10 as “major” predictor. Overall, the performances in terms of C-index and HR of the PI in stratifying risk groups remained moderate: as an example, the C-index of the models for the validation set, reported in Table 3, ranged between 0.590 and 0.625. On the other hand, the potential in stratifying patients according to risk classes is evident if looking to Figure 1 and to the Figure S2 in the Supplementary Materials, outperforming, to our knowledge, existing predictive markers.

**Figure 2:**
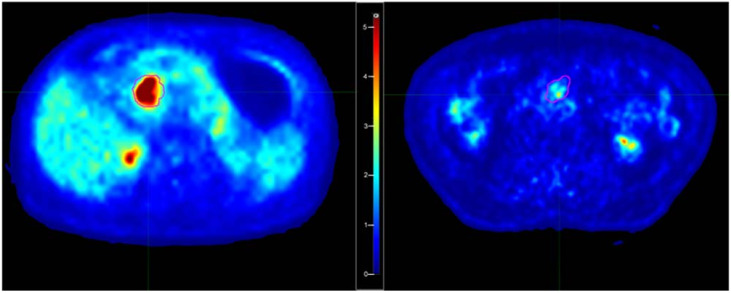
Examples of patients with “high” (**left**) and “low” (**right**) Percentile10 values of the segmented tumor. The corresponding SUV values for Percentile10 were respectively 7.62 and 1.41.

Few considerations may be made regarding the meaning of the selected features; a higher value of Percentile10 is associated with an increased risk of early distant relapse. This finding was already discussed by Mori et al. [57]: a higher value of this feature is consistent with lesions with a deep uptake, showing a ‘‘compact” aspect, with little blurring at the edges. Furthermore, the corresponding intensity histograms are characterized by a more shifted shape versus higher intensities. As an example, in Figure 2, the central axial images of two patients with “high” and “low” Percentile10 values are shown. This feature is also strongly associated with Statistical_minGreylevel which was retained as one of the best-predicting features in Model 3. Not surprisingly, when forcing the model to include Percentile10 (Model 4), Statistical_minGreylevel was replaced by Percentile10. On the other hand, the other retained features (Intensity_Histogram_coefficientofVariation, GLSZM3D_glVariance, COMShift) are all related to signal heterogeneity within the lesion. For all three cases, the coefficients of the models indicate that the risk increased for a lower heterogeneity, corroborating (and likely refining) the information of “image compactness” captured by Percentile10. Then, overall, a deep and relatively homogeneous uptake seems to be associated with a more aggressive tumor in terms of rapidity in the spreading of distant relapses.

Very importantly, the prognostic indexes derived here are expected to have good generalizability due to the reduced number of robust features, with a major role of first-order features. The absence of complex features in the model is an advantage in terms of inter-scanner and inter-center variability. The replication of their performances outside our center is the focus of the second part of the currently ongoing trial related to these analyses: the collection of images and data is already in an advanced phase, and first results should be available within a short time.

The confirmation of the value of the suggested few-features models would be of great value, and the positive results of current temporal validation are promising. The emphasis on distant recurrence stems from the fact that metastatic spread is the most common form of recurrence and progression in LAPC, as well as the leading cause of death. Early identification of patients with a low probability of metastasis through a radiomic signature could (1) facilitate new clinical studies and improve therapeutic outcomes by intensifying treatments for patients with a better prognosis, and/or (2) reduce overtreatment for patients with a poorer prognosis, significantly enhancing the therapeutic approach.

Importantly, the value of local intensification of the treatment is still in part controversial [19,24,26]: the translation of reducing local relapses into a gain in overall survival is clearly largely modulated by the individual risk of early distant relapses. In a recent study from our group [17], it was shown how the group of patients who can benefit from local intensification, for instance through dose escalation delivered with advanced image-guided methodologies/technology [14,29,30,31,32], is quite limited. Moreover, local intensification cannot be yet considered as without risks, due to the proximity of very sensitive organs at risk [27]. The identification of imaging biomarkers, such as the ones here suggested, could better support the exploration of intensified approaches based on rational, personalized patient selection. More research in this direction is warranted.

## 5. Conclusions

The analysis conducted on a large population of patients with inoperable LAPC revealed that PET radiomic features can effectively predict distant recurrence-free survival (DRFS) after chemoradiotherapy. The model demonstrated good discriminative ability in both the training and validation samples. Further validation studies on independent cohorts from other centers are currently underway to confirm these findings.

As the final aim of the RADIOMIPa project, a clinical trial will be proposed based on the resulting prognostic index in the selection of the patients to be treated with local treatment intensification: the modality of local intensification (such as the choice of dose, volumes, and dose fractionation) has yet to be discussed.

The link between radiomic features and tumor biology is an emerging and increasingly relevant field, owing to the greater availability of imaging data compared to costly and complex molecular analyses. While invasive tissue sampling remains the gold standard for histological characterization, non-invasive imaging techniques could offer a valuable alternative or complement, particularly for patients with advanced or inoperable disease, as demonstrated in this study.

## Figures and Tables

**Table 1:** Characteristics of the population under study. Starting from the left: the first column lists the features under examination; the second column shows the values for the training population; the third column displays the numbers for the validation population. Finally, the rightmost column contains the *p*-values obtained from the comparison of the two populations using the Mann–Whitney test. In this column, features where the test revealed a statistically significant difference between the two groups (*p*-value < 0.05) are marked with an asterisk (*).

	Train	Validation	MW *p*-Value
N° of patients	145	70	
Sex			
Male	76 (52.41%)	30 (42.86)	0.190
Female	69 (47.59%)	40 (57.14%)	
Age (median; range) (y)	64.41 [44–85]	66.55 [35–87]	0.925
Tumor side			
Body	15 (10.35%)	6 (8.57%)	0.684
Head	44 (30.35%)	35 (50%)	0.005 *
Tail	1 (0.69%)	2 (2.86%)	0.207
Head-body	34 (23.45%)	15 (21.43%)	0.742
Body-tail	12 (8.27%)	5 (7.14%)	0.775
Missing	39 (26.89%)	7 (10%)	0.005 *
Histology			
Adenocarcinoma	119 (82.07%)	63 (90%)	0.132
Cystoadenocarcinoma	2 (1.38%)	0 (0%)	0.328
Missing	24 (16.55%)	7 (10%)	0.202
Tumor stage			
III	129 (88.97%)	60 (85.71%)	0.495
IV	16 (11.03)	10 (14.29)	
Distant progression (DRFS)			
Yes	90 (62.07%)	41 (58.57%)	0.624
No	55 (37.93%)	29 (41.43%)	
Time of DRFS (median, range) (m)	6.88 [0–33]	7.52 [0.3–39.3]	0.909
Local Progression (LRFS)			
Yes	74 (51.03%)	35 (50%)	0.888
No	42 (28.97%)	21 (30%)	0.877
Missing	29 (20%)	14 (20%)	1.0
Time LRFS (median; range) (m)	14.9 [4.76–46.96]	14.92 [1.35–28]	0.697
Overall Survival (median; range) (m)	18.7 [4.76–51.83]	21.06 [7.87–42.33]	0.923
Follow-up (mean; range) (m)	13.04 [0.35–166.8]	15.33 [0.71–59.29]	0.605
Scanner PET			
Discovery—ST	31 (21.38%)	7 (10%)	0.041 *
Discovery—STE	84 (57.93%)	49 (70%)	0.089
Discovery—690	29 (20%)	14 (20%)	0.328
Other	2 (1.38%)	0 (0%)	1.0

**Table 2:** The first three major rows present the best three models obtained from the analysis of RFs. Specifically, the Mori model is the one obtained by Mori et al. [57]; Model 2 considers only the predominant RF from the Mori model. Model 3 was obtained using the 1000 bootstrap populations method with the MedicalAI software. For these three models, the table reports the coefficient b with its *p*-value, the model’s *p*-value, the C-index, the Hazard Ratio (HR), and the Logrank test *p*-value obtained from Kaplan–Meier (KM) separation, both on the training population and the validation population. These values are provided both for the pre-harmonization analysis and for the post-harmonization analysis using the ComBat method. Finally, at the bottom of the table, Model 4 obtained using 1000 bootstrap populations with RFs harmonized via the ComBat method is reported.

	Before Harmonization						After Harmonization					
Variable *	Coeff.	*p*-Value Var.	*p*-Value Model	C-Index	HR	*p*-Value KM	Coeff.	*p*-Value Var.	*p*-Value Model	C-Index	HR	*p*-Value KM
**Mori model**												
**Train**												
Morphological COMshift	−0.235	0.098	0.0009	0.603	1.85	0.005	−0.298	0.026	0.0005	0.607	2.03	0.002
Statistical Percentile 10	1.42 × 10^−4^	0.0015					1.35 × 10^−4^	0.0043				
**Validation**												
PI **	0.49	0.126	0.12	0.55	1.72	0.107	0.525	0.12	0.11	0.554	2.07	0.028
**Model 2**												
**Train**												
Statistical Percentile 10	1.52 × 10^−4^	0.0007	0.0011	0.605	2.53	0.0002	1.46 × 10^−4^	0.0023	0.0033	0.6	1.81	0.007
**Validation**												
PI **	0.644	0.059	0.0522	0.543	1.7	0.12	0.743	0.046	0.042	0.551	1.67	0.128
**Model 3**												
**Train**												
Statistical min grey level	1.97 × 10^−4^	0.0004	0.0008	0.589	2.83	0.0001	2.08 × 10^−4^	0.0009	0.0012	0.592	3.01	< 0.0001
Intensity histogram coeff. of Variation	−3.86	0.0099					−4.042	0.008				
**Validation**												
PI **	0.75	0.0232	0.021	0.592	1.87	0.049	0.83	0.012	0.0103	0.603	1.97	0.0415
**Model 4**												
**Train**												
Statistical Percentile 10							1.89 × 10^−4^	0.0001	0.001	0.641	4.86	< 0.0001
GLSZM3D grey level variance							−8.29 × 10^−3^	0.0104				
**Validation**												
PI **							0.385	0.0588	0.042	0.581	2.15	0.0276

* The meaning of the radiomic features that appear in these models are presented in the Supplementary Materials. ** Prognostic Index.

**Table 3:** The four models considered are reported, to which the clinical variable of tumor stage has been added. This variable is dichotomous (1 for those with stage IV and 0 for those with stage III). The first three major rows present the best three models obtained from the analysis of RFs. Specifically, the Mori model is that obtained by Mori et al. [57]; Model 2 considers only the predominant RF from the Mori model. Model 3 was obtained using the 1000 bootstrap populations method with the MedicalAI software. For these three models, the table reports the coefficient b with its *p*-value, the model’s *p*-value, the C-index, the Hazard Ratio (HR), and the Logrank test *p*-value obtained from Kaplan– Meier (KM) separation, both on the training population and the validation population. These values are provided for both the pre-harmonization analysis and the post-harmonization analysis using the ComBat method. Finally, at the bottom of the table, Model 4 is reported, which was obtained using 1000 bootstrap populations with RFs harmonized via the ComBat method.

	Before Harmonization						After Harmonization					
Variable *	Coeff.	*p*-Value Var.	*p*-Value Model	C-Index	HR	*p*-Value KM	Coeff.	*p*-Value Var.	*p*-Value Model	C-Index	HR	*p*-Value KM
**Mori model**												
**Train**												
Morphological COMshift	−0.266	0.0793	0.0002	0.633	3.02	< 0.0001	−0.293	0.057	0.002	0.632	2.88	<0.0001
Statistical Percentile 10	1.86 × 10^−4^	0.003					1.79 × 10^−4^	0.0003				
Grading	0.5001	0.107					0.508	0.102				
**Validation**												
PI **	0.581	0.0636	0.068	0.582	1.74	0.18	0.63	0.0485	0.053	0.59	13.42	0.0188
**Model 2**												
**Train**												
Statistical Percentile 10	2.02 × 10^−4^	0.0001	0.0004	0.624	2.20	0.001	1.94 × 10^−4^	0.0001	0.0004	0.619	2.26	0.0024
Grading	0.431	0.161					0.433	0.159				
**Validation**												
PI **	0.616	0.0514	0.057	0.0575	2.13	0.074	0.694	0.0348	0.0396	0.601	1.67	0.2103
**Model 3**												
**Train**												
Statistical min grey level	2.69 × 10^−4^	<0.0001	0.0002	0.619	2.76	0.0001	2.62 × 10^−4^	0.0001	0.0002	0.617	3.27	<0.0001
Intensity histogram coeff. of Variation	−4.624	0.0043					−4.79	0.0035				
Grading	0.560	0.0706					0.572	0.0655				
**Validation**												
PI **	0.622	0.021	0.026	0.625	2.44	0.043	0.637	0.0063	0.0077	0.625	2.6	0.0417
**Model 4**												
**Train**												
Statistical Percentile 10							2.36 × 10^−4^	<0.0001	0.0001	0.654	4.48	<0.0001
GLSZM3D grey level variance							−8.29 × 10^−3^	0.0172				
Grading							0.402	0.1936				
**Validation**												
PI **							0.597	0.0150	0.0072	0.623	2.34	0.0488

* The meaning of the radiomic features that appear in these models are presented in Supplementary Materials. ** Prognostic Index.

## Data Availability

No data are publicly available due to Ethical Committee constraints.

## Supplementary Materials

The following supporting information can be downloaded at: https://www.mdpi.com/article/10.3390/cancers17061036/s1, The supplementary materials include the robust radiomic features (Tables S1 and S2), their significance, their correlation (Table S3), and the intra-scanner statistical comparison (Table S4). The bootstrap technique is then briefly presented (Figure S1). Finally, all the models that were considered are presented (Table S5 and Figure S2).
