# Supplementary material for: Temporal Validation of an FDG-PET-Radiomic Model for Distant-Relapse-Free-Survival After Radio-Chemotherapy for Pancreatic Adenocarcinoma
Source: Cancers (Basel). 2025 Mar 20;17(6):1036. doi: 10.3390/cancers17061036 (PMC11941493; doi:10.3390/cancers17061036)
Supplement: Supplementary file 1 [file cancers-17-01036-s001.zip › cancers-3504224-supplementary.pdf]

Supplementary Materials

On the following pages, supplementary materials are provided. Specifically, the radiomic features will be explained (how they were selected and their significance), the bootstrap method used to obtain the models will be described, and finally, all the models under consideration will be presented.

S1. Radiomic Features (RF)

S1.1: Robustness of Radiomic Features

As in the previous study conducted by Mori et al. [59] and considering the results of Presotto [69] and Belli [51], we included only the robust RF in the analysis, which were not impacted by either intra-scanner variability or intra-operator variability.

Starting from the 182 RF extracted with SPAARC, 80 RF influenced by intra-observer variability were discarded. Additionally, 35 RF (listed in Table S1) were excluded, as the Mori study identified them as being strongly affected by inter-scanner variability and image acquisition/reconstruction. Cox modeling codes from medicalAI were then applied to the remaining 78 RF. Of these, 60 RF were further discarded due to redundancy and correlation. The final 18 RF, used for bootstrapping, are listed in Table S2, with the RF included in the models shown in bold.

Table S1: The RF found by Mori et al. [59] to be strongly influenced by inter-scanner variability (first block) and acquisition/reconstruction parameters (second block).

	RF FAMILY	RF NAME
**RF influenced by inter-scanner variability**	GLCM3D_avg	Joint Variance
	GLCM3D_comb	Joint Variance
	GLSZM3D	Small Zone Emphasis
	GLSZM3D	Large Zone High Grey Level Emphasis
	GLDZM3D	Large Distance high Grey Level Emphasis
	GLDZM3D	Zone Distance Non Uniformity Norm
	NGLDM3D*	Low Dependence Emphasis
	NGLDM3D*	Hight Dependence Emphasis
	NGLDM3D*	Low Dependence High Grey Level Emphasis
	NGLDM3D*	High Dependence High Grey Level Emphasis
	NGLDM3D*	Grey Level Non Uniformity
	NGLDM3D*	Dependence Count Non Uniformity
	NGLDM3D*	Dependence Count Non Uniformity Norm
	NGLDM3D*	Grey Level Variance
	NGLDM3D*	Dependence Count Variance
	NGLDM3D*	Dependence Count Entropy
	NGLDM3D*	Dependence Count Energy
**RF influenced by image acquisition/reconstruction.**	GLCM3D_avg	Correlation
	GLCM3D_comb	Correlation
	GLRL3D _avg	Short Run Emp
	GLRL3D _avg	Long Run Emp
	GLRL3D _avg	Run Percentage
	GLRL3D _avg	Grey level Variance
	GLRL3D _avg	rlVariance
	GLRL3D_comb	Short Run Emp
	GLRL3D_comb	Long Run Emp
	GLRL3D_comb	Run Percentage

GLRL3D_comb	Grey level Variance
GLRL3D_comb	rlVariance
GLSZM3D	Large Zone Emphasis
GLSZM3D	Zone Size Variance
GLDZM3D	Grey Level Variance
NGTDM3D	Coarseness
NGTDM3D	Complexity
NGTDM3D	Strength

* RF excluded from the new version of SPAARC.

Table S2: List of the 18 final RF resulting from medicalAI, on which models were obtained using the bootstrap technique. The ones that were then included in the models are in bold.

RF FAMILY	RF NAME
Morphology	Area Density convexHull
Morphology	COMshift
Morphology	Vol Density aee
Morphology	Elongation
Morphology	Flatness
Morphology	Area Density aabb
Morphology	Vol Density aabb
Statistical	Percentile 10
Statistical	Kurtosis
Statistical	Min Grey Level
Intensity Histogram	Coefficient of Variation
GLCM3D_comb	Info Correlation 2
GLCM3D_comb	Cluster Shade
GLCM3D_avg	Cluster Prominence
GLDZM3D	Low Grey Level Zone Emphasis
GLDZM3D	Large Distance high Grey Level Emphasis
GLSZM3D	Grey Level Variance
NGTDM3D	Contrast

S1.2: Significance of Radiomic Features

In this section, the meanings of the radiomic features included in the various models under consideration are provided.

Morphology_COMshift: This feature quantifies the displacement of a lesion's center of mass (COM) relative to its geometric center in a 3D image. Essentially, it measures how much the lesion’s actual "centroid" deviates from its ideal center, assuming a regular shape. A *COMShift* value of 0 indicates that the COM perfectly coincides with the geometric center, suggesting a symmetrical and regular lesion shape; values greater than 0 indicate that the COM is shifted from the geometric center, suggesting an asymmetrical or irregular lesion shape. Very high values might indicate a highly irregular and asymmetrical lesion.

Statistical_percentile10: This represents the 10th percentile of the intensity distribution within a Region of Interest (ROI). In other words, it indicates the intensity value below which 10% of the voxels within the ROI are found. A low value suggests that most of the voxels within the ROI have relatively low intensity, while a high value suggests that most voxels have relatively high intensity.

Statistical_variance: This represents the variance of intensity values within an ROI. Simply put, it provides an indication of how much the intensity values within the ROI deviate from the mean. A high *Variance* indicates that the intensity values within the ROI vary significantly from each other, while a low *Variance* indicates that the intensity values are quite similar to each other.

Statistical_minGreylevel: This indicates the minimum intensity value within an ROI. In other words, it represents the voxel with the lowest intensity within the ROI.

Intensity_Histogram_coefficientofVariation: This is a measure of the heterogeneity of intensity values within a ROI in a medical image. Essentially, this feature indicates how much the intensity values vary from each other within the ROI. A high value indicates a large variability in intensity values within the ROI, which could be associated with heterogeneous tissues, the presence of lesions, or other characteristics causing a non-uniform distribution of intensity values. A low value suggests low variability in intensity values, indicating more homogeneous tissues or a more uniform region of interest.

GLSZM3D_glVariance (Gray Level Size Zone Matrix 3D – gray level Variance): This measures the variance of the grey-level intensity within the image. A high value indicates that grey-level intensity varies greatly throughout the volume, while a low value indicates that grey-level intensity is more consistent.

GLDZM3D_smallDistanceEmphasis (Gray Level Distance Zone Matrix 3D): This measures the emphasis on co-occurrences between nearby pixels in terms of intensity and distance in the 3D grey-level distance zone matrix. A high value indicates that there are many co-occurrences of pixels with similar intensity values that are spatially close to each other.

S1.3: Correlations between the RFs of the various models

This section presents the Spearman correlation coefficients resulting from the MedicalAI analysis.

Table S3: Features coefficient of Spearman correlation.

	Morphology COMshift	Statistical percentile10	Statistical variance	Statistical min grey level	Intensity Histogram coefficient of Variation	GLDZM3D small distance emphasis	GLSZM3D grey level variance
**Morphology COMshift**	1	-0,156	0,403	-0,307	0,232	-0,422	-0,240
**Statistical percentile10**	-0,156	1	0,602	0,918	0,194	0,099	0,354
**Statistical variance**	0,403	0,602	1	0,380	0,503	-0,328	0,136
**Statistical min grey level**	-0,307	0,918	0,380	1	0,236	0,286	0,448
**Intensity Histogram coefficient of variation**	0,232	0,194	0,503	0,236	1	0,105	0,383
**GLDZM3D small distance emphasis**	-0,422	0,099	-0,328	0,286	0,105	1	0,398
**GLSZM3D grey level variance**	-0,240	0,354	0,136	0,448	0,383	0,398	1

S1.4: Intra-Scanner Statistical Significance

Below are the 11 RFs that showed statistically significant differences (p-value < 0.05) in the intra-scanner analysis based on the Mann-Whitney test.

Table S4: Features and scanners showing statistically significant differences (Mann-Whitney test p-value < 0.05) before harmonization via ComBat.

Features	Scanner 1	Scanner 2	p-value
Morphology area Density aabb	Discovery STE	Discovery 690	0,012290

Morphology volume Density aee	Discovery STE	Discovery 690	0,007309
Statistical kurtosis	Discovery STE	Discovery ST	0,013085
Statistical min Grey Level	Discovery STE	Discovery ST	0,000619
Statistical min Grey Level	Discovery 690	Discovery ST	0,033295
Statistical percentile10	Discovery STE	Discovery ST	0,001152
Statistical percentile10	Discovery 690	Discovery ST	0,018947
Intensity Histogram coefficient of Variation	Discovery STE	Discovery 690	0,004595
Intensity Histogram coefficient of Variation	Discovery 690	Discovery ST	0,003445
GLCM3D avg cluster Prominence	Discovery STE	Discovery ST	0,008315
GLCM3D comb infoCorrelation2	Discovery STE	Discovery 690	0,060124
GLCM3D comb infoCorrelation2	Discovery STE	Discovery ST	0,057433
GLSZM3D grey level Variance	Discovery STE	Discovery ST	0,068573
GLDZM3D low Grey Level Zone Emphasis	Discovery STE	Discovery 690	0,015119
GLDZM3D low Grey Level Zone Emphasis	Discovery 690	Discovery ST	0,052515
GLDZM3D large Distance high Grey Level Emphasis	Discovery STE	Discovery 690	0,000058
GLDZM3D large Distance high Grey Level Emphasis	Discovery STE	Discovery ST	0,002138

Section SM2: Bootstrap technique

The bootstrap method is widely used in the computational aspects of medical physics, as it is one of the primary resampling techniques, alongside cross-validation [85]. In this study, it was employed to generate multiple scenarios based on real data from clinical images, with the goal of identifying a more stable and reliable model.

The procedure starts with an original sample of size n. A new sample, referred to as a "bootstrap sample," is created by randomly selecting data points with replacement from the original sample, meaning that each data point can be selected multiple times or not selected at all. This is depicted in Figure S1. In this case, 1000 bootstrap samples (or populations) were generated.

For each bootstrap sample, the desired statistical measures are calculated, resulting in a distribution of statistics from the various bootstrap samples. The final model is selected based on two key criteria: it should exhibit the highest frequency of occurrence across the bootstrap populations and a high C-index, which indicates its predictive power and generalizability.

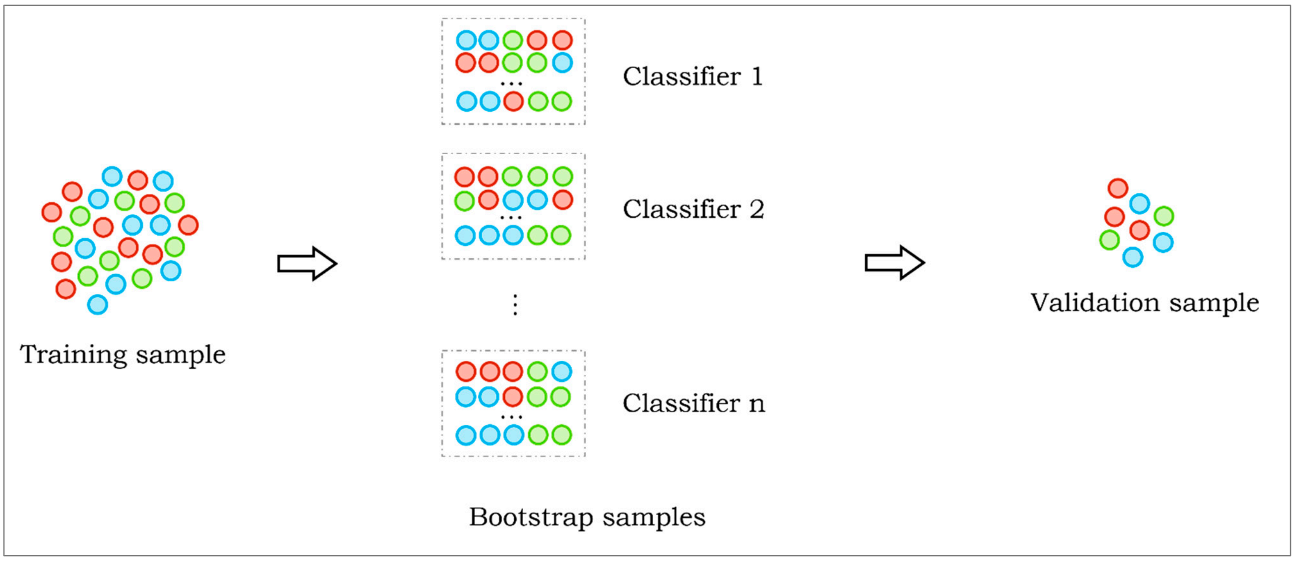

Figure S1: graphical representation of the bootstrap technique. The image was created by the author for illustrative purposes in this study.

Section SM3: Considered Models

Before harmonization with Combat, five models were considered: (i) the model by Mori et al. [59], (ii) a univariate model with the strongest RF from the previous model; (iii) a two-variable model obtained through cross-validation; (iv) a two-variable model and (v) a three-variable model obtained through 1000 bootstrap populations. After feature harmonization, the same five models were re-evaluated. These models, both pre- and post-harmonization, are detailed in Table S5. The model resulting from the post-harmonization analysis is presented in Tables 2 and Table 3 of the main text.

Below the table, the Kaplan-Meier separations are reported for the models presented in the main body (Table 3), incorporating radiomic variables and the clinical staging variable.

Table S5: The five models considered are reported. Model A is taken from the paper by Mori et al. [59]; Model B incorporates the strongest RF from Model A. Model C was found using the cross-validation method with the population divided into 5 folds. Model D was identified through the 1000 bootstrap population method. Model E was obtained with 1000 bootstrap populations, but considering 3 predictive variables instead of two like model D. Finally, Model F is the two-variable model obtained after harmonization via ComBat.

	Before harmonization						After harmonization					
**Variable**	**Coeff.**	**p-value var.**	**p-value model**	**C-index**	**HR**	**p-value KM**	**Coeff.**	**p-value var.**	**p-value model**	**C-index**	**HR**	**p-value KM**
**Model A: original or Mori model**												
**Train**												
Morphological COMshift	-0.235	0.098	0.0009	0.603	1.85	0.005	-0.298	0.026	0.0005	0.607	2.03	0.002
Statistical Percentile 10	1.42 E-04	0.0015					1.35 E-04	0.0043				
**Validation**												
PI*	0.49	0.126	0.12	0.55	1.72	0.107	0.525	0.12	0.11	0.554	2.07	0.028
**Model B**												
**Train**												
Statistical Percentile 10	1.52 E-0.4	0.0007	0.0011	0.605	2.53	0.0002	1.46 E-04	0.0023	0.0033	0.6	1.81	0.007
**Validation**												
PI*	0.644	0.059	0.0522	0.543	1.7	0.12	0.743	0.046	0.042	0.551	1.67	0.128
**Model C: cross-validation**												
**Train**												
Statistical Percentile 10	1.91 E-04	0.0001	0.0014	0.605	2.12	0.0007	1.97 E-03	0.0005	0.0037	0.602	2.49	0.0001
Statistical Variance**	-2.7 E-08	0.15					-2.7 E-08	0.13				
**Validation**												
PI*	0.703	0.027	0.022	0.578	2.12	0.032	0.865	0.014	0.0105	0.596	2.02	0.028
**Model D: bootstrap**												
**Train**												
Statistical min grey level	1.97 E-04	0.0004	0.0008	0.589	2.83	0.0001	2.08 E-04	0.0009	0.0012	0.592	3.01	<0.0001
Intensity histogram coeff. of Variation	-3.86	0.0099					-4.042	0.008				
**Validation**												
PI*	0.75	0.0232	0.021	0.592	1.87	0.049	0.83	0.012	0.0103	0.603	1.97	0.0415
**Model E: bootstrap 3 variables**												
**Train**												
Statistical min grey level	2.24 E-04	0.0002	0.0038	0.615	2.51	0.0001	2.29 E-04	0.0006	0.0055	0.617	2.24	0.0003
GLDZM3D small distance emphasis	-3.015	0.0121					-3.17 E-08	0.096				
Statistical Variance	-2.7 E-08	0.16					-3.33	0.0096				
**Validation**												

PI*	0.642	0.0486	0.0334	0.586	2.09	0.0535	0.738	0.0307	0.0189	0.602	2.66	0.0181
**Model F: bootstrap after ComBat harmonization**												
**Train**												
Statistical Percentile 10							1.89 E-04	0.0001	0.001	0.641	4.86	<0.0001
GLSZM3D grey level variance							-8.29 E-03	0.0104				
**Validation**												
PI*							0.385	0.0588	0.042	0.581	2.15	0.0276

*Prognostic Index
** Correlation with COMshift: 0.4

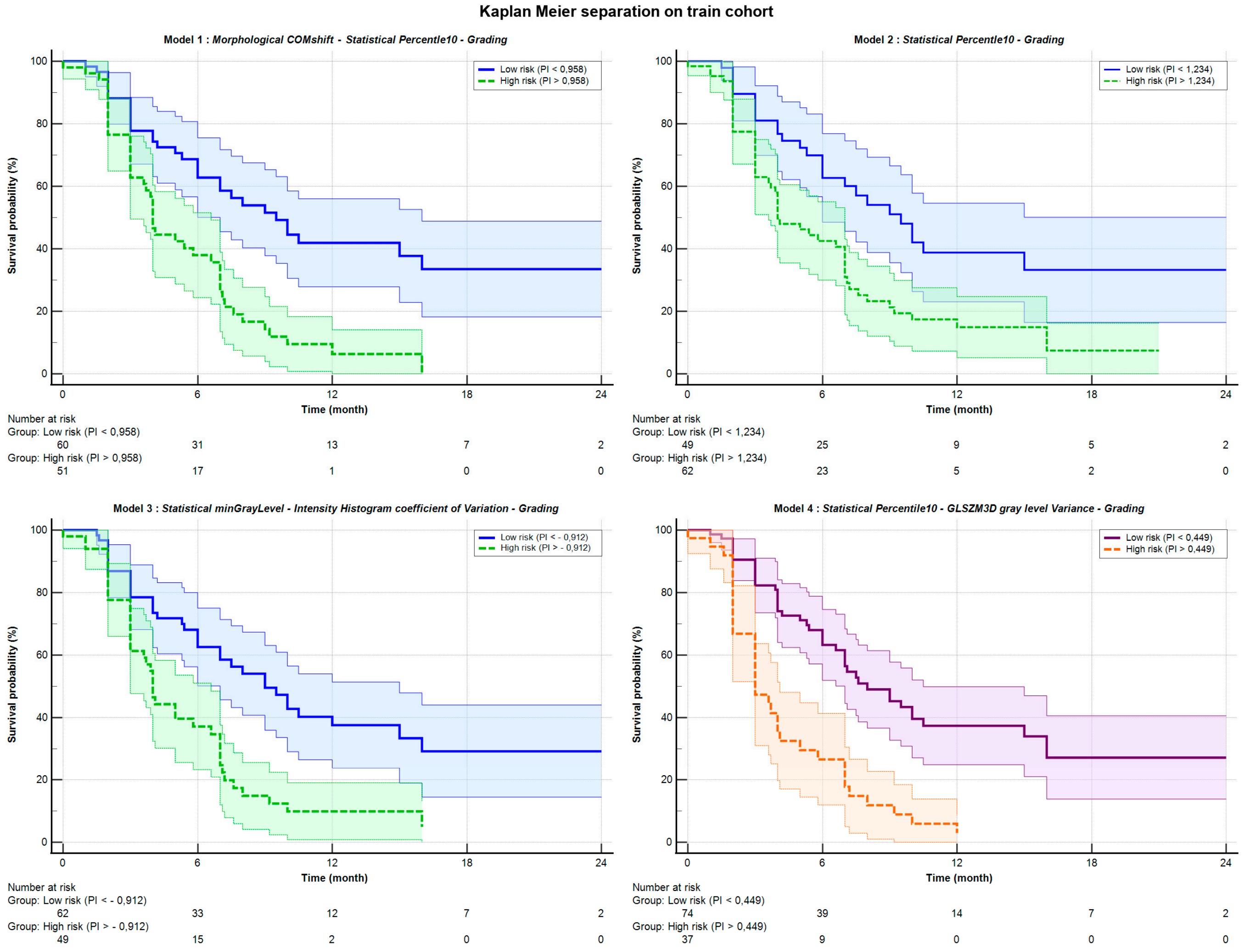

(a)

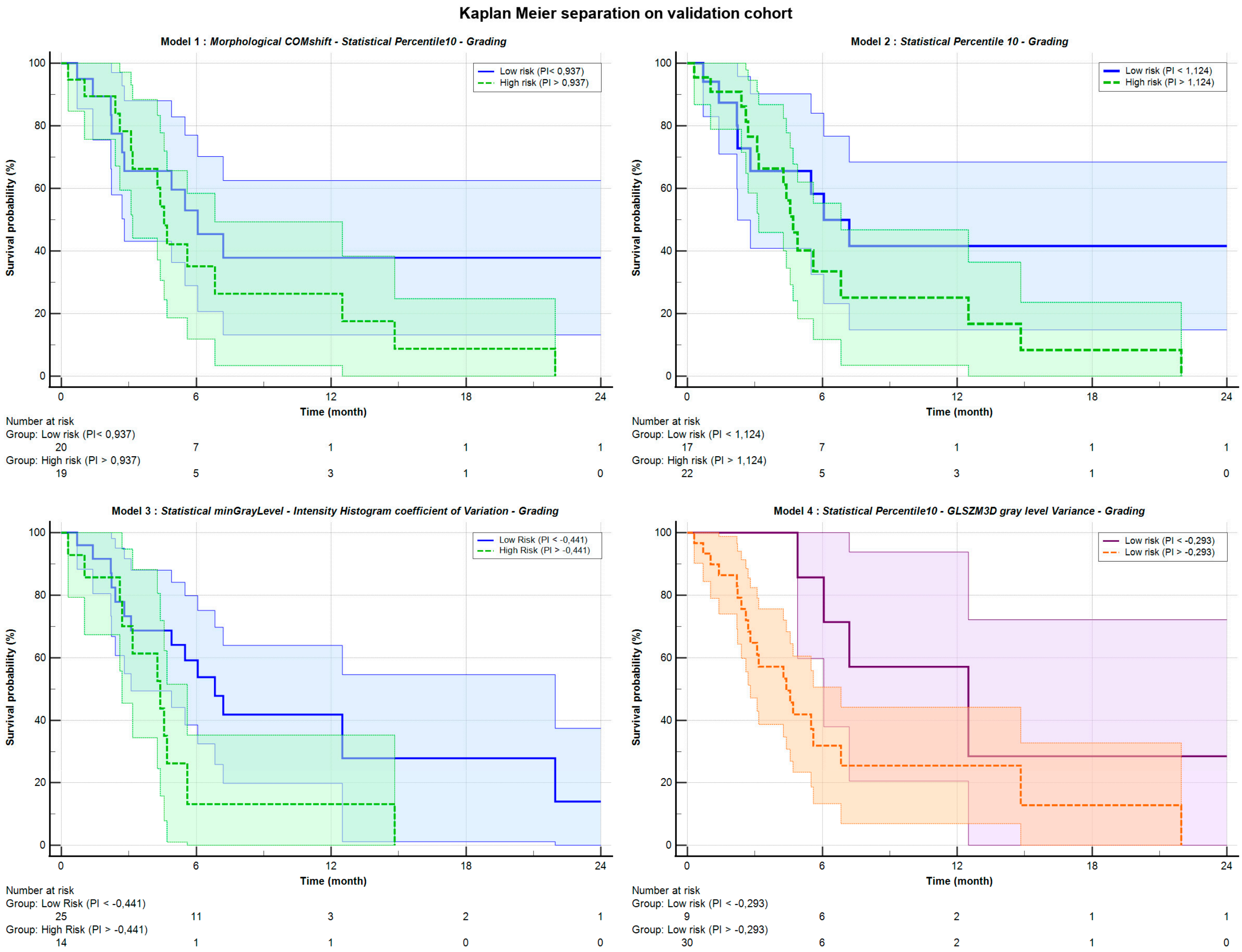

(b)

Figure S2: Kaplan-Meier separation curves for training **(a)** and validation **(b)** cohort with corresponding confidence bands, considering both radiomic features and clinical grading variable as predictive factors (values in Table 3 of the main text). The solid blue and purple line represent the curve for low probability, while the dashed green and orange line represent the high-risk curve. The separation was performed based on the Youden index obtained from ROC analysis. Starting from the top left, the separations are shown for Model 1 (COMshift, Percentile10 and Grading), Model 2 (Percentile10 and Grading) and Model 3 (minGrayLevel, coefficient of Variation and Grading). In the bottom right corner, Model 4 (comprising Percentile10, gray level Variance, and Grading) is displayed, obtained after harmonization with the ComBat method.
